# Adaptation strategies of leaf traits and leaf economic spectrum of two urban garden plants in China

**DOI:** 10.1186/s12870-023-04301-z

**Published:** 2023-05-24

**Authors:** Liying Xu, Nana Zhang, Tongchao Wei, Bingyang Liu, Lanyi Shen, Yang Liu, Dounan Liu

**Affiliations:** 1 School of Life Science and Technology, Mudanjiang Normal College, Mudanjiang, Heilongjiang 157011 P. R. China; 2 School of Chemistry and Chemical Engineering, Mudanjiang Normal College, Mudanjiang, Heilongjiang 157011 P. R. China; 3 Key Laboratory of Sustainable Forest Ecosystem Management-Ministry of Education, School of Forestry, Northeast Forestry University, Harbin 150040 P. R. China

**Keywords:** Climate, Life form, Leaf morphology traits, Leaf physiological traits, Correlation

## Abstract

**Background:**

Previous studies of the relationships between traits have focused on the natural growth conditions of wild plants. Urban garden plants exhibit some differences in plant traits due to environmental interference. It is unknown whether the relationships between the leaf traits of urban garden plants differ under distinct climates. In this study, we revealed the variation characteristics of the leaf functional traits of trees, shrubs, and vines in two urban locations. Two-way ANOVA was used to reveal the response of plant leaf traits to climate and life forms. Pearson correlation analysis and principal component analysis were used to calculate the correlation coefficient between the leaf functional traits of plants at the two locations.

**Results:**

Leaf dry matter content (LDMC) and vein density (VD) of different life forms in Mudanjiang were higher than those in Bozhou (*P* < 0.05), and the relative water content (RWC) in Bozhou was higher, whereas vein density (VD) of trees and shrubs in the two urban locations was significant (*P* < 0.05), but the vines were not significant. The photosynthetic pigments of tree and shrub species were larger in Mudanjiang, but the opposite was true for the vines. Both leaf vein density (VD) and stomatal density (SD) showed a very significant positive correlation in the two urban locations (*P* < 0.01), and both were significantly positively correlated with specific leaf area (SLA) (*P* < 0.05); and negatively correlated with leaf thickness (LT), and the relationship between pigment content were closer.

**Conclusion:**

The response to climate showed obvious differences in leaf traits of different life forms species in urban area, but the correlations between the traits showed convergence, which reflects that the adaptation strategies of garden plant leaves to different habitats are both coordinated and relatively independent.

**Supplementary Information:**

The online version contains supplementary material available at 10.1186/s12870-023-04301-z.

## Introduction

Leaves connect plants and the environment; and bridge connecting plants and ecosystems [1, 2]. The leaf structural traits (e.g., specific leaf area, leaf thickness, stomatal density, leaf vein density, and leaf dry matter content) and physiological traits (e.g., photosynthetic rate and pigment) can reflect the adaptive strategies of plants to climate [3–8]. The leaf traits, which are key attributes of plant growth status, reflect the adaptation and response of plants to the growth environment [9–10]. The differences of leaf traits are affected by many factors (e.g., interspecific, community, longitude and latitude, etc.) [9, 11–13]. All of these studies are based on studies of plants growing naturally in the wild. With the intensification of urbanization, differences in the leaf functional traits of urban garden plants under the same climatic conditions have been studied deeply [14–16]. Zhang studied 36 plants species in Lingering Garden and showed that the leaf thickness (LT) and leaf dry matter content (LDMC) of trees were higher than shrubs, whereas specific leaf area (SLA) of shrubs was higher than that of trees, and leaf area (LA), leaf volume (LV) and leaf tissue density (LTD) were not significantly different in distinct life form species [17]. Moreover, the trait characteristics of different life forms are affected by various abiotic environmental factors such as water and light [18]. Therefore, it is necessary to investigate leaf traits of urban garden plants of different life forms under distinct climatic conditions.

Wright et al. studied leaf trait (A_mass_, R_mass_, LMA, LL, etc.) data of 2548 plant species from 175 regions worldwide and proposed the concept of “leaf economics spectrum” (LES) for the first time, which depicts a series of interlinked and synergistic functional trait combinations on a global scale, and quantifies and outlines a series of plant resource trade-off strategies that vary in a regular and continuous manner. At one end of the LES are leaf functional traits with small LMA, short leaf lifespan, high leaf N content, high photosynthetic rate, and high dark respiration rate, which represent " --Quick investment-return species” type of plant survival strategy, whereas the other end of the spectrum is the “Slow investment-return” type with large LMA, long leaf lifespan, high leaf N content, low photosynthetic rate, and low dark respiration rate [19]. In recent years, some studies have found that relationships between plant leaf traits are prevalent in plant populations, communities, and biota [19–23], suggesting that leaf tissue construction requires a trade-off between plant growth and resource acquisition [4, 24]. Principal component analysis (PCA) has proven to be an effective approach for analyzing the comprehensive impacts of environmental factors [25–27]. Maire and Asner showed that the relationships between leaf economic spectrum traits have different response patterns and similar or different trade-offs within and among habitats, suggesting that environmental factors and their own genetic traits affect leaf trait relationships in plants [28, 29]. Although the leaf traits of urban garden plants have some differences due to environmental interference, it is unknown whether the relationships among urban garden plant leaf traits differ under distinct climates.

**Fig. 1:**
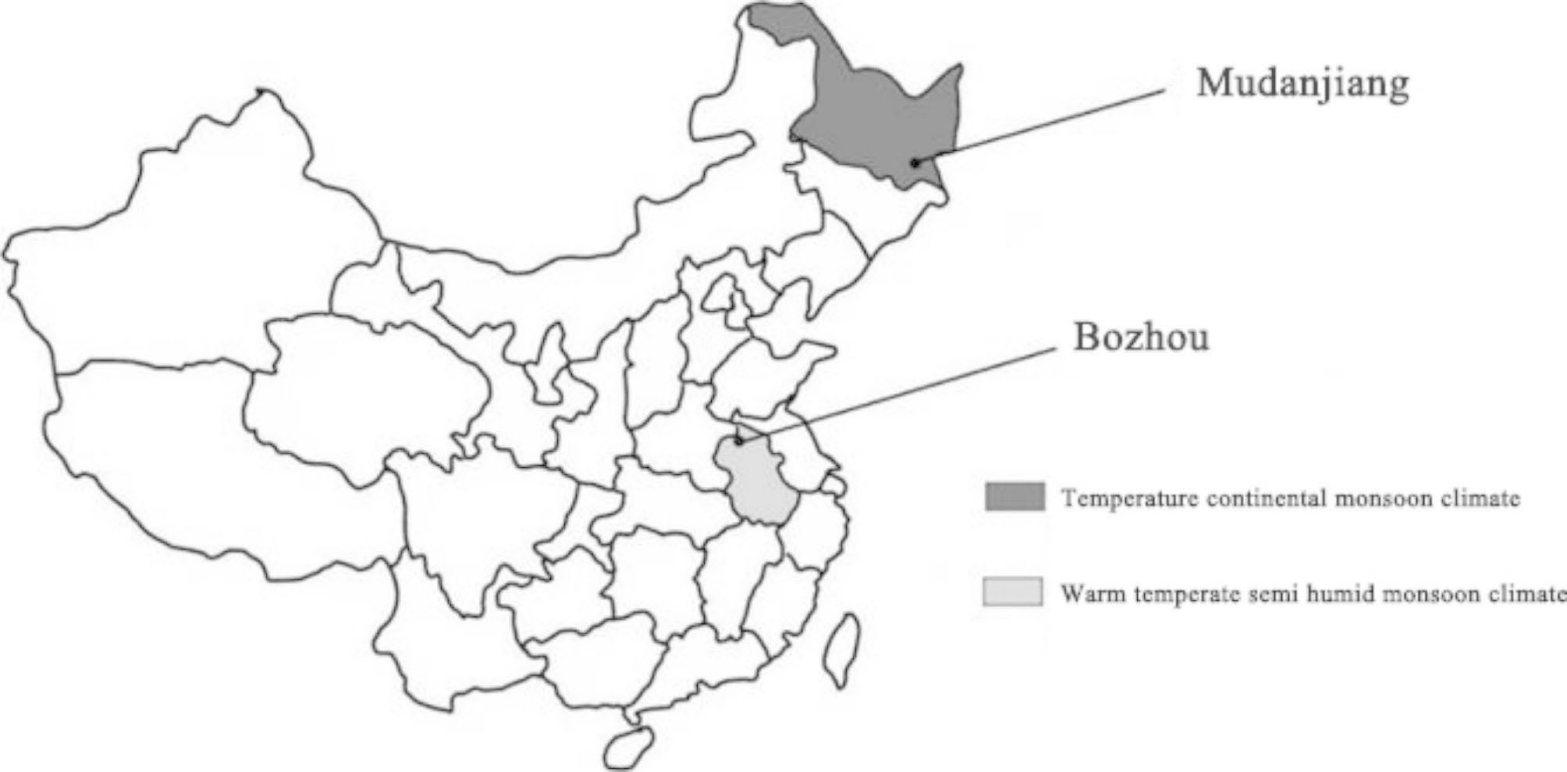
Geographical location of the study area

In this study, we sampled leaves from two climatic zones with different precipitation, temperatures and altitudes (Fig. 1, Table S1) as the research object (Table 1), we sampled common woody plant leaves on campus (17 species in Mudanjiang, Heilongjiang Province, and 9 species in Bozhou, Anhui Province). For each species, we measured 11 functional traits of the plant leaves, covering their morphological and physiological characteristics (Table 2). Our goal was to explore the variation in leaf traits of urban garden plants with different life forms in the two cities and the correlation of leaf traits at the species level to reveal the adaptive strategies adopted by plants in the two cities to adapt to different climates. We tested two hypotheses: (1) There are differences in the response of leaf traits to climate among species and different life forms and (2) The LES is widespread [30], which is not affected by climate change. Studying the changes and correlations of these attributes in species and life forms can help us to understand the ecological adaptation strategies of urban plants with different life forms under different climates. This study provides a scientific basis for the selection and application of urban garden plants.

**Table 1:** List of species studied, family and life form

Site	Species	Family	Genus	Life form
Mudangjiang	*Ulmus pumila* L cv ‘Jinye’	Ulmaceae	Ulmus	Tree
	*Acer saccharum* Marsh.	Aceraceae	Acer	Tree
	*Acer pictum*Thunb. ex Murray	Aceraceae	Acer	Tree
	*Acer mandshuricum* Maxim.	Aceraceae	Acer	Tree
	*Betula platyphylla* Suk.	Betulaceae	Betula	Tree
	*Prunus cerasifera* f. *atropurpurea*	Rosaceae	Prunus	Tree
	*Padus virginiana* ‘Canada Red’	Rosaceae	Padus	Tree
	*Sorbus pohuashanensis*	Rosaceae	Sorbus	Tree
	*Spiraea thunbergii* Bl.	Rosaceae	Spiraea	Shrub
	*Spiraea x bumalda* cv.Gold Flame	Rosaceae	Spiraea	Shrub
	*Berberis thunbergii* DC.	Berberidaceae	Berberis	Shrub
	*Acer ginnala* Maxim.	Aceraceae	Acer	Shrub
	*Physocarpus opulifolius* var.*luteus*	Rosaceae	Physocarpus	Shrub
	*Physocarpus opulifolius ‘Summer Wine’*	Rosaceae	Physocarpus	Shrub
	*Parthenocissus quinquefolia* (L.) Planch.	Vitaceae	Parthenocissus	Vine
	*Parthenocissus tricuspidata*	Vitaceae	Parthenocissus	Vine
	*Vitis vinifera* L	Vitaceae	Vitis	Vine
Bozhou	*Acer pictum*Thunb. ex Murray	Aceraceae	Acer	Tree
	*Prunus cerasifera* f. *atropurpurea*	Rosaceae	Prunus	Tree
	*Ginkgo biloba* L.	Ginkgoaceae	Ginkgo	Tree
	*Acer palmatum* Thunb.	Aceraceae	Acer	Tree
	*Loropetalum chinense* var. *rubrum*	Hamamelidaceae	Loropetalum	Shrub
	*Nandina domestica* Thunb.	Berberidaceae	Nandina	Shrub
	*Photinia × fraseri* Dress	Rosaceae	Photinia	Shrub
	*Parthenocissus tricuspidata*	Vitaceae	Parthenocissus	Vine
	*Vitis vinifera* L.	Vitaceae	Vitis	Vine

## Results

### Variation in plants leaf traits in two areas

The descriptive statistics of the 11 leaf traits of the plants in the two areas showed that the traits varied widely (Table 2). The degree of variation in the pigment content of the 17 plants was larger than that in the morphological traits in Mudanjiang. The largest variation was in Chlb (118.06%) and the smallest was in LMDC and LT (19.33%). In Bozhou, the largest variation among the 9 plants was in SLA (127.95%) and the smallest variation was in RWC (10.40%). Leaf morphological traits were significantly affected by climate except for LT whereas pigment content was not significantly affected. LA, LDMC, and RWC were significantly affected by life forms and the interactions between climate and life forms (*P* < 0.001) (Table 3).

**Table 2:** Leaf trait parameters and variation characteristics of plants in two urban locations in China. Abbreviations: LT: Leaf thickness; LA: Leaf area; LDMC: Leaf dry matter content; RWC: Leaf relative water content; SLA: Specific leaf area; SD: Stomatal density; VD: Leaf vein density; Chla: Chlorophyll a; Chlb: Chlorophyll b; Car: Carotinoid; Chl: Chlorophyll

Urban in China	Leaf trait	Mean ± SD	Min	Max	Kurtosis	Skewness	CV(%)
Mudangjiang	LT	0.17 ± 0.03	0.11	0.24	-0.21	0.23	19.33
	LA	25.48 ± 25.91	3.06	119.74	12.39	3.31	101.70
	LDMC	0.61 ± 0.12	0.34	0.85	1.05	-0.19	19.33
	RWC	0.49 ± 0.14	0.26	0.81	0.71	0.40	27.40
	SLA	235.25 ± 72.83	127.55	406.54	0.61	0.66	30.96
	SD	218.32 ± 122.11	75.90	584.79	4.29	1.77	55.93
	VD	5.91 ± 1.48	3.38	9.17	0.31	0.29	25.11
	Chla	4.37 ± 5.11	0.22	17.58	1.45	1.43	116.96
	Chlb	1.19 ± 1.40	0.01	4.68	1.36	1.48	118.06
	Car	2.43 ± 1.17	0.52	5.32	1.02	0.71	47.96
	Chl	5.56 ± 6.49	0.26	22.27	1.45	1.45	116.85
Bozhou	LT	0.17 ± 0.03	0.13	0.21	-1.53	-0.16	15.92
	LA	29.23 ± 37.39	3.15	124.12	6.67	2.49	127.95
	LDMC	0.38 ± 0.83	0.23	0.48	-0.94	-0.53	22.29
	RWC	0.75 ± 0.08	0.63	0.85	-0.84	-0.34	10.40
	SLA	145.58 ± 47.81	77.10	203.17	-1.79	-0.13	32.84
	SD	354.36 ± 109.41	210.61	559.29	0.13	0.58	30.87
	VD	3.56 ± 1.23	1.44	5.10	-0.34	-0.60	34.70
	Chla	4.43 ± 2.81	1.22	9.04	-1.28	0.43	63.46
	Chlb	1.00 ± 0.86	0.17	2.17	-2.20	0.39	86.41
	Car	1.10 ± 0.65	0.55	2.57	2.92	1.62	59.35
	Chl	5.42 ± 3.54	1.57	10.53	-1.94	0.30	65.26

**Table 3:** Two-way analysis of variance (ANOVA) for the effects of climatic (Mudangjiang; Bozhou), life form (Tree; Shrub; Vine), and their significant interaction to plant morphological traits and physiological traits in China. Abbreviations: LT: Leaf thickness; LA: Leaf area; LDMC: Leaf dry matter content; RWC: Leaf relative water content; SLA: Specific leaf area; SD: Stomatal density; VD: Leaf vein density; Chla: Chlorophyll a; Chlb: Chlorophyll b; Car: Carotinoid; Chl: Chlorophyll

Variables	*df*	*P values*										
		LT	LA	LDMC	RWC	SLA	SD	VD	Chla	Chlb	Car	Chl
Climatic (Cl)	1	0.550	0.037	**0.001**	**0.002**	**0.001**	**0.032**	**0.008**	**0.921**	0.802	0.062	0.983
Life form(Lf)	2	0.058	**0.004**	**0.029**	**0.019**	0.317	0.382	0.132	0.302	0.224	0.108	0.285
Cl×Lf	1	0.547	**0.023**	**0.047**	**0.022**	0.171	0.542	0.361	0.550	0.994	0.320	0.638

Note: p-values in bold indicate significant effects. Non-significant interaction effects are not displayed

### Plants leaf traits of different life forms in two areas

**Fig. 2:**
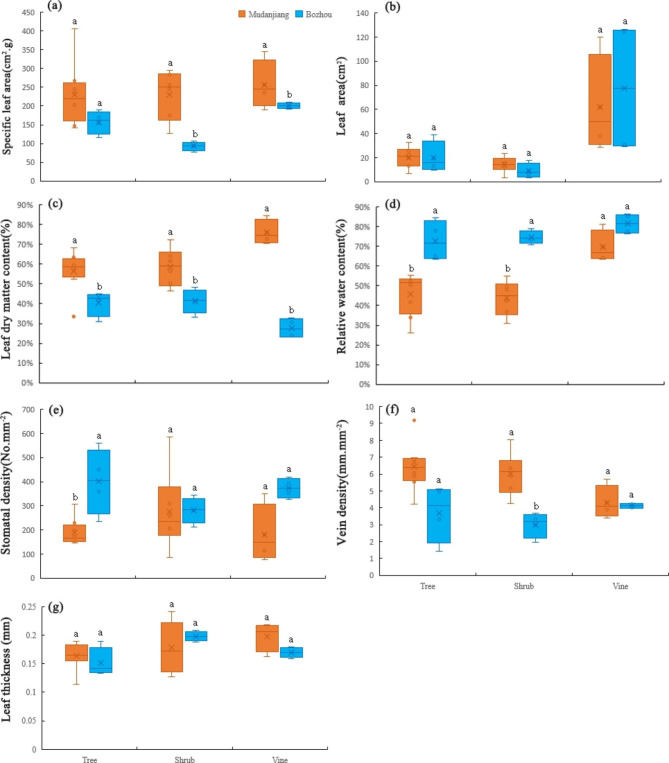
Specific leaf area(cm^2^·g^− 1^) (**a**), Leaf area (cm^2^) (**b**), Leaf dry matter content(%) (**c**); Relative water content(%) (**d**), Stomatal density(No ·mm^− 2^) (**e**), Leaf vein density(mm·mm^− 2^) (**f**), Leaf thickness (mm) (**g**) of tree, shrub and vine in two urban locations in China. Different lowercase letters indicate significant differences at *P* < 0.05 levels

The leaf morphological traits of the two areas were different (Table S2; Fig. 2). The SLA, LDMC, VD and LT of trees, shrubs, and vines in Mudanjiang were significantly higher than those in Bozhou (Fig. 2a,c,f,g), whereas the LA, RWC, and SD showed the opposite trend. The SLA of trees, shrubs, and vines in Mudanjiang were higher than those in Bozhou at 46.87%, 147.35%, and 27.78%, respectively, and the RWC was lower than those of Bozhou at 36.96%, 41.39%, and 14.46%, respectively (Fig. 2a, d). In terms of life forms, the SLA, LA and RWC of vines were the highest and those of shrubs were the lowest in both areas. LT was in the order of shrubs > vines > trees and VD was in the order of trees > shrubs, but SD was inconsistent. The leaf pigment contents of different life forms also differed between the two sites (Table 4). The pigment contents of trees and shrubs in Mudanjiang were higher than those in Bozhou, whereas the pigment contents of vines showed the opposite trend. The pigment contents of shrubs and vines differed significantly between the two areas, except for that of Car. The largest difference is in the Chlb of shrubs, which was 6.67 times higher in Mudanjiang than in Bozhou. The Car of trees in the two areas differed significantly and that in Mudanjiang was twice as much as that in Bozhou. In terms of life forms, the pigment content of all plants in Mudanjiang showed shrubs > trees > vines, whereas the opposite was shown in Bozhou.

### Correlation between leaf traits in two areas

**Fig. 3:**
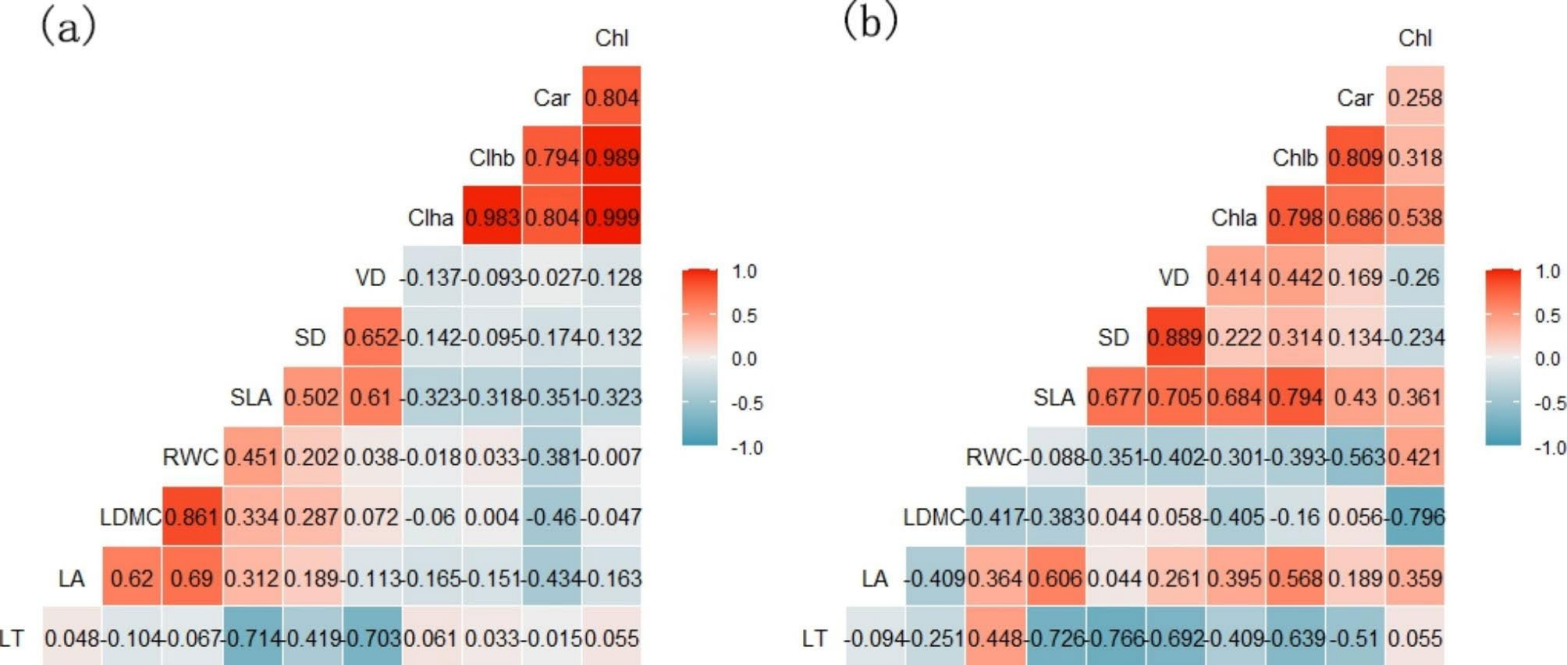
Correlation between plant leaf traits in two urban locations in China. Abbreviations: LT: Leaf thickness; LA: Leaf area; LDMC: Leaf dry matter content; RWC: Leaf relative water content; SLA: Specific leaf area; SD: Stomatal density; VD: Leaf vein density; Chla: Chlorophyll a; Chlb: Chlorophyll b; Car: Carotinoid; Chl: Chlorophyll. (**a**): Mudangjiang; (**b**): Bozhou

There were significant positive and negative correlations between the various plant leaf morphological traits in the two areas (Fig. 3). There was a significantly negative correlation between LT and SLA in both areas (*R*^*2*^ = 0.527, *P* < 0.05, *R*^*2*^ = 0.533, *P* < 0.001). Compared with Mudanjiang, there was a stronger positive correlation between VD and SD in Bozhou (*R*^*2*^ = 0.372, *P* < 0.01, *R*^*2*^ = 0.791, *P* = 0.01). The SLA in the two areas showed significant positive correlations with VD and SD, and the correlation between SLA and VD was stronger in Bozhou (*R*^*2*^ = 0.587, *P* < 0.05). There were significant negative correlations between LT and VD in both areas. There was a significant negative correlation between LT and SD in Bozhou (*R*^*2*^ = 0.587, *P* < 0.05). We found positive correlations between various pigments in the two areas, except for the correlation between Car and Chl in Bozhou. However, the positive correlation between Chla and Chlb was stronger in Mudanjiang (*R*^*2*^ = 0.966, *P* < 0.001).

### Principal component analysis of leaf traits in the two study areas

**Fig. 4:**
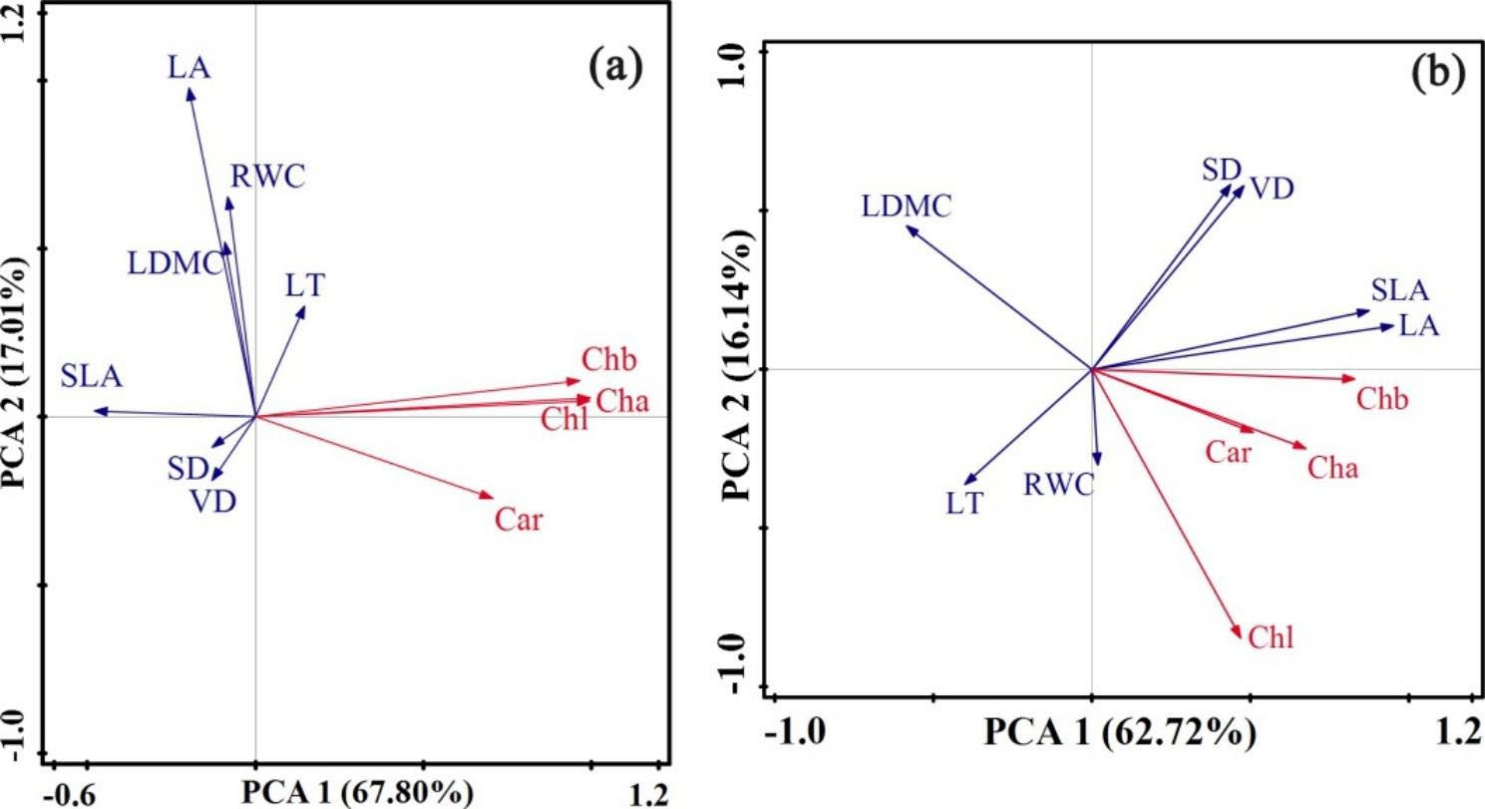
Principal component analysis of species leaf morphological traits (blue lines) and physiological traits (red line) in two urban locations in China. Abbreviations: LT: Leaf thickness; LA: Leaf area; LDMC: Leaf dry matter content; RWC: Leaf relative water content; SLA: Specific leaf area; SD: Stomatal density; VD: Leaf vein density; Chla: Chlorophyll a; Chlb: Chlorophyll b; Car: Carotinoid; Chl: Chlorophyll.(**a**): Mudangjiang; (**b**): Bozhou

Principal component analysis showed that the first and second sequence axes of the two areas cumulatively explained 84.81% and 78.86% of the total variation in leaf traits, respectively (Fig. 4a, b).The first axis of Mudanjiang explained 67.80% of the variation in leaf traits and was negatively correlated with SLA, LA, SD, VD, LDMC, and RWC and positively correlated with Chl, Chla, Chlb, Car, and LT. The second sequence axis explained 17.01% of the variation in leaf traits and was positively correlated with LA, RWC, LDMC, LT, Chlb, Chl, Chla, and SLA and negatively correlated with Car, VD, and SD. The first sequence axis (62.72%) and second sequence axis (16.14%) of Bozhou explained the total variation in the leaf traits. The first axis was negatively correlated with LDMC, LT, and RWC and positively correlated with LA, SLA, Chlb, Chla, Chl, Car, VD, and SD. The second axis was positively correlated with VD, SD, LDMC, SLA, and LA and negatively correlated with Chl, LT, RWC, Chla, Car, and Chlb.

**Table 4:** Pigment contents of tree, shrub and vine in two urban locations in China

Life form	Site	Chlorophyll a (mg g^− 1^)	Chlorophyll b (mg g^− 1^)	Carotenoids (mg g^− 1^)	Chlorophyll (mg g^− 1^)
Tree	Mudangjiang	4.03 ± 1.72a	1.14 ± 0.50a	2.55 ± 0.30 a	5.17 ± 2.21a
	Bozhou	3.9 ± 1.49a	1.2 ± 0.50a	1.29 ± 0.48 b	5.1 ± 1.96a
Shrub	Mudangjiang	6.35 ± 2.57a	1.64 ± 0.70a	2.9 ± 0.58 a	7.99 ± 3.26a
	Bozhou	2.88 ± 0.62b	0.25 ± 0.05b	0.73 ± 0.09 b	3.12 ± 0.60b
Vine	Mudangjiang	1.31 ± 0.41b	0.41 ± 0.07b	1.18 ± 0.33 a	1.72 ± 0.48b
	Bozhou	7.8 ± 1.24a	1.71 ± 0.22a	1.25 ± 0.11 a	9.51 ± 1.02a

Note: Different lowercase letters indicate significant differences at P < 0.05 levels

## Discussion

The variation in plant traits is influenced by the environment and symbiotic organisms [31]. Plants can produce similar traits in local habitats, whereas the divergence traits of plants are produced by competition. There was ecological niche differentiation in species coexistence, which reflected the mechanism of species coexistence. In this study, there were different degrees of variation in leaf traits in 17 and 9 garden plant species in the two areas. The average variation which was 61.78% (Mudanjiang) and 49.95% (Bozhou), reflected the response of species to environmental changes during community construction [32], that proved our first hypothesis. The highest variations in all the plant traits in the two areas were in LA and pigment content. Bozhou showed greater variation in LA, and Mudanjiang showed greater variation in pigment content. According to the climate of the two locations, the mean annual sunshine of the two locations are similar, but the average elevation of Mudanjiang is higher, while the mean annual temperature and mean annual precipitation are lower than that of Bozhou. It can be seen that the environmental conditions with higher temperature and precipitation had a greater impact to LA, while the environment with higher elevation had a greater impact on photosynthetic pigments. This suggests that LA and pigment content are more sensitive to environmental changes and that it exhibits greater phenotypic plasticity in such environment [33]. In addition, LT and RWC are relatively stable along the resource axis because the coefficients of variation of the two traits are small in these two areas [10].

### Effects of climate on leaf morphological traits

Leaves are the main organs of plants for assimilation and transpiration and are the most sensitive organs to the environment [34, 35]. Plants have evolved their own adaptive traits over a long evolutionary period. The 6 leaf traits of the plants in this study were significantly different between the two climatic conditions, indicating that the morphological building process differs when plant leaves adapt to the environment [36]. In general, a lower SLA indicates a strong ability to use environmental resources and preserve obtained resources [37]. Therefore, it is more suitable for arid and barren environments. The larger SLAs are likely to have higher light-capture capacities and relative growth rates. In this study, the SLA of shrubs and vines in Mudanjiang was significantly higher than those in Bozhou, which was in the range of Quick investment-return species [19]. This is probably due to the lower light intensity in Mudanjiang, which tends to increase plant SLA [38]. In addition, it is speculated that the plants in this area may have a low capacity of environmental resources and a low capacity of the obtained resources [39]. LDMC is as a relatively stable prediction index on the resource acquisition axis. A higher LDMC indicates that plants make better use of the environmental resource conditions of their habitat [40]. The LDMC of all life forms in Mudanjiang was significantly higher than that in Bozhou (*P* < 0.05). The LDMC of plants increases with decreasing temperature [41], because a higher LDMC can prevent plants from losing water and nutrients. LDMC is a common indicator of water retention in leaves. It is an important indicator of plant traits that reflects the ability of plants to withstand drought environments [42]. The LDMC of trees and shrubs in Bozhou was significantly higher than in Mudanjiang with an average of 30.09%. The average rainfall in Bozhou is 1.5 times higher than that of Mudanjiang. It is possible that the more water the leaves absorb, the stronger the water retention capacity of the leaves, so that the LDMC is significantly larger.

### Effects of climate on leaf physiological traits

Chlorophyll plays a crucial role in photosynthesis and is the basic component of the membrane material in plant organs. It is a vital indicator of photosynthetic capacity, nutritional status, and growth trends in plants [43–45]. Generally, Chla is considered to be the central pigment of photosynthesis, its concentration can be used to monitor plant growth and characterize external interference [46, 47]. Chlb transmits light energy to Chla that can boost the efficiency of plant utilization of light energy. Previous studies had shown that chlorophyll content of plant leaves decreases with increasing altitude [48], while others had suggested that it increases with altitude [49]. In this study, the pigment content of trees and shrubs was greater in Mudanjiang than in Bozhou, which suggesting that these life forms plants had higher photosynthetic capacity in the climate condition with higher altitudes, lower temperatures and less rainfall, while the opposite was for vines, which reflecting the plant response to photosynthesis physiological characteristics and adverse environmental conditions. The vines in the Bozhou had a larger LA and a sufficiently strong photosynthetic capacity, this may be the main reason why chlorophyll content of vines had a significantly greater than those in Mudanjiang. It can be seen that plants of different life form have developed their own photosynthetic characteristics in a stable growth environment. Car is a very important auxiliary pigment in chloroplasts, capturing light energy and transferring it to Chla for photosynthesis. Mudanjiang, as the high altitudes, where light radiation is strong, plants can protect themselves from light damage by increasing their carotenoid content [50].

### Interrelations of leaf traits in two areas

During the process of evolution and adaptation to the environment, plants have formed a series of trait combinations that are optimal for adapting to the environment [51]. LES provides a new theory and method for analyzing plant adaptation mechanisms [19]. In this study, there was a significant negative correlation between LT and SLA (Bozhou *P* < 0.001, Mudanjiang *P* < 0.001), SD and VD were positively correlated with SLA and negatively correlated with LT in both the climates. This finding confirms our second hypothesis, many researches support these opinions [17, 22, 52]. This shows that the coordination of plant structure and function converges in different environments. This study verified the existence of LES under different climatic conditions. In summary, when applying the theory of leaf economic spectrum in this study, we need to fully consider the community type, its own development, plant functional type and life form, environmental conditions, disturbance factors, and so on. The differences in these conditions may result in different leaf economic spectra [53–56].

In addition, our study found that the correlation relationship between LA and LDMC showed inconsistency in the two locations, while Pang studied of 18 plants in Southwest Guangxi and Zhang studied of 36 plants in the Liuyuan Garden both found that LA showed a negative correlation with LDMC [17, 57], indicating the instability of the relationship between LA and LDMC. We analysed the climatic conditions in these locations and found that, the relatively temperature in Mudanjiang are lower, plants need to increase LA to supplement light energy in order to achieve leaf dry matter accumulation [58]. The correlation between SLA and pigment content was higher in Bozhou, Oren found that the photosynthetic capacity of *Picea abies* diminished with decreasing SLA in adult species [59], while Cheng found that pigment content could be added to LES traits [60]. It can be seen that it is necessary to include of photosynthetic pigment indicators in LES studies.

Principal component analysis showed that the PCA1 axis was negatively correlated with LDMC and RWC, and positively correlated with pigment content. The PCA2 axis was positively correlated with SLA, LDMC, and LA and negatively correlated with Car (Fig. 4). This indicated that there were some differences in the balance strategies of plant leaf traits under different climates, but these balance strategies had smaller differences in pigments. In our study, there was no correlation between leaf traits and climate. This is also the main research direction for the future.

## Conclusion

In this study, the differences and relationships between leaf traits were investigated. All leaf traits were based on plants of different life forms in two areas with different water, altitude, and light intensity. We found that the leaf traits of 17 species of garden plants in Mudanjiang and 9 species of garden plants in Bozhou varied to different degrees. The SLA, LDMC, VD, and LT of trees, shrubs, and vines in Mudanjiang were relatively high, whereas LA, RWC, and SD in Bozhou were higher. The pigment contents of the shrubs Mudanjiang and the vines in Bozhou were higher. The difference in the number of trees between the two areas was not significant. This indicated that the sensitive responses to the climate of different life forms of leaf traits were different. These results provide a basis for the selection of urban garden plants in two urban locations. In addition, there was a relationship between morphological traits and pigment content of leaves at the two locations, but there were also some differences. The relationships between the pigments were close to each other. This reflects the differences and coordination of the balance strategies of the leaf traits.

## Methods

### Study site

Two study sites were chosen from the south-north environmental gradient in China. Mudanjiang is located in the southeast of Heilongjiang Province, China. The terrain is mainly mountainous and hilly. The altitude ranges from 300 to 800 m, with an average of 230 m. The soil is mainly dark brown. Spring short, quick to warm up, windy, and easy to drought; summer warm and rainy; autumn short and quick to cool down; winter long and cold. Rainfall is mainly concentrated in summer and is characterized by simultaneous rain and heat [61]. Bozhou is influenced by meandering and cutting changes in rivers and the successive southern flooding of the Yellow River. A plain is formed in which posts, slopes, and saucer-shaped depressions are distributed among each other, with the geomorphological characteristics of “big flat and small uneven”. The soil is mainly sandy concretionary black soil, followed by tidal soil, and brown soil with little lime soil. Bozhou has a mild climate, sufficient sunlight, moderate rainfall, and a long frost-free period with an average of 216 days. The four seasons in Bozhou are distinct: spring temperature is changeable, summer rain is concentrated, autumn is crisp, and winter is long and dry [62]. The locations and climatic characteristics of the two cities are shown in Table S1 and Fig. 1, respectively.

### Plot establishment and sampling

We selected common local garden plants in the two cities in late September, 2020 (Table 1). Seventeen woody plant species (8 species of trees, 6 species of shrubs, and 3 species of vines) were selected from the campus of Mudanjiang Normal College, and 9 species of woody plants (4 species of trees, 3 species of shrubs, and 2 species of vines) were selected from the campus of Lixin County No. 1 Middle School. All voucher specimens were deposited in the herbarium of Mudanjiang Normal College. At each site, 6 standard plants of each species were randomly selected for leaf sampling and the tree height and diameter at breast height (DBH) were recorded. Sixty sunny leaves were collected for each plant. The leaf samples were divided into two subsamples: one was gently washed with deionized water and immediately fixed in formalin-aceto-alcohol (FAA) solution (90 ml of 50% ethanol, 5 ml of 100% glacial acetic acid and 5 ml of 37% formaldehyde) for leaf vein analysis and the other was placed in Ziploc bags for morphological and physiological analyses. The selected leaves were packed and stored in a portable refrigerator and transported to the laboratory.

### Morphological traits measurement

In the laboratory, the leaf samples in each Ziploc bag were cleaned with distilled water, and absorbed the water with filter paper. We weighed 5 groups of leaves (6 leaves/group) on a balance and recorded the fresh leaf weight as W1. The samples were then placed in distilled water for more than 6 h to absorb water and reach saturation. Every leaf was removed and dried to remove any water residue on the surface, weighed, and the saturated fresh weight was recorded as W2. Then dried to constant weight at 65 °C to obtain the leaf dry mass (accuracy = 0.0001 g) and the dry weight was recorded as W3. The leaf dry matter content (LDMC) was calculated as follows:

LDMC = W3/W1.

The relative water content (RWC) was calculated as follows:

RWC= (W1-W3)/(W2-W3).

Leaf thickness was measured as far as possible to avoid the main veins of the leaves and secondary veins on both sides using a Vernier caliper (0.02 mm).We measured 3 times for 1 group/plant (6 leaves) and with repetition of 5 groups/plants and then calculated the average thickness of each leaf (leaf thickness, LT). The leaves of 5 plants/groups used for leaf morphological analysis were scanned with a scanner (dpi = 400, Epson Telford Ltd, Telford, UK) and then dried to a constant weight at 65 °C to obtain the leaf dry mass. The scanned images of the leaves were processed using Image-J 136b (National Institutes of Health, MD, USA) to obtain the leaf area. Specific leaf area (SLA) was calculated as leaf area divided by leaf dry mass. Stomatal density was measured by nail polish impression method as discribed in Franks [63]. A transparent nail polish was uniformly applied to the leaf abaxial surface, allowing it to harden, and clear cellophane tape was used to transfer the impression of the stomata to a microscope slide. The stomatal properties were observed under a compound microscope (BX-51, Olympus Corporation, Tokyo, Japan). Three images were selected from each leaf, and 45 observation fields for 15 leaves of each species were counted and measured. The stomatal density (SD) was calculated as the stomatal number divided by the observation field area. The leaves were cut into 1 cm × 1 cm pieces, placed in vials with FAA fixative, and soaked in 5% NAOH for 4–5 days (the soaking solution was changed daily). We removed and placed the leaves on slides when they became transparent, covered them with coverslips, and used the method described by Poethig [64]. Three images were selected from each leaf, and 45 observation fields for 15 leaves of each species were counted and measured. We then measured the total length of the leaf vein using MIPlus software. Leaf vein density (VD) was calculated as the total length of the leaf vein divided by the observation field area.

### Leaf physiological traits measurement

Referring to the mixed solution extraction method of Cang [65], leaves (0.5 g) were cut into tubes containing 80% acetone. The leaves were extracted in the dark for 12 h and photosynthetic pigments content were measured at 662 nm, 644 nm and 440 nm using a visible spectrophotometer (INESA, model L3S).

The calculation of Chlorophyll content;

Chla = 12.21OD_663_-2.81OD_646_

Chlb = 20.13OD_646_-5.03OD_663_

Car= (1000OD_470_-3.27C_a_-104C_b_)/229

Chlorophyll content (Chl) (mg g^-1^) = N × Vt × C/W

where C is the concentration of pigment (mg•L^− 1^); Vt is the volume of extract (ml); N is the dilution multiple, W is the fresh weight of the sample (g).

### Data analysis

The descriptive statistical analysis was performed for each trait in both areas. The coefficient of variation (CV = standard deviation/mean × 100%) was used to characterize the degree of variation for each trait. Two-way ANOVA was used to calculate the effects of climate and life forms on each trait, and one-way ANOVA was used to calculate the mean and standard error of each trait for the leaves of trees, shrubs, and vines in the two areas. Before calculation, all data were tested for normal distribution using the chi-square tests. Differences between treatments were tested using Duncan (D) if the hypothesis was met, otherwise differences between treatments were tested using Dunnett^,^ s T3 (LSD, α = 0.05). Pearson correlation analysis and principal component analysis were performed to explore the interrelationships between and within the traits of the plants in the two areas. Pearson’s correlation analysis was calculated using the ‘corrplot’ package in ‘R’ [66]. Microsoft Excel 2003 and SPSS software (version 19.0, SPSS Inc., Cary, NC, USA) were used to process data. Microsoft Excel 2003 and Origin 2021 were used to plot data.

## Electronic supplementary material

Below is the link to the electronic supplementary material.

Supplementary Material 1

## Data Availability

All data generated or analyzed during this study are included in this article and additional information is available from the authors upon request.
