# Supplementary material for: Adaptation strategies of leaf traits and leaf economic spectrum of two urban garden plants in China
Source: BMC Plant Biol. 2023 May 24;23:274. doi: 10.1186/s12870-023-04301-z (PMC10207826; doi:10.1186/s12870-023-04301-z)
Supplement: Supplementary file 1 — Supplementary Material 1 [file 12870_2023_4301_MOESM1_ESM.docx]

**Table S1.** Sampling sites and climatological data of two climatic regions

| Site | Longitude | Latitude | Average  elevation (m) | Mean annual temperature(℃) | Mean annual precipitation(mm) | Mean annual sunshine(h) | Landform |
| --- | --- | --- | --- | --- | --- | --- | --- |
| Mudangjiang | E129°35' | N44°36' | 230 | 5.9 | 547 | 2305 | Mountains, Hhills |
| Bozhou | E116°21' | N33°14' | 28 | 14.7 | 822 | 2320 | Plain |

**Table S2.** Leaf trait of 17species in Mudanjiang and 9 species in Bozhou in China. Abbreviations: LT: Leaf thickness; LA: Leaf area; LDMC: Leaf dry matter content; RWC: Leaf relative water content; SLA: Specific leaf area; SD: Stomatal density; VD: Leaf vein density; Chla: Chlorophyll a; Chlb: Chlorophyll b; Car: Carotinoid; Chl: Chlorophyll.

| Site | Species | LT | LA | SLA | SD | VD | RWC | LDMC | Chla | Chlb | Car | Chl | LT |
| --- | --- | --- | --- | --- | --- | --- | --- | --- | --- | --- | --- | --- | --- |
| Mudangjiang | *Ulmus pumila* L cv ‘Jinye’ | 0.166 | 6.853 | 203.922 | 154.017 | 4.226 | 0.261 | 0.336 | 1.141 | 0.010 | 2.633 | 1.151 | 0.166 |
|  | *Acer saccharum* Marsh. | 0.175 | 22.110 | 267.892 | 230.085 | 6.898 | 0.554 | 0.633 | 0.275 | 0.146 | 1.004 | 0.421 | 0.175 |
|  | *Acer pictum*Thunb. ex Murray | 0.160 | 32.633 | 234.211 | 158.889 | 6.064 | 0.339 | 0.523 | 0.220 | 0.042 | 2.161 | 0.262 | 0.160 |
|  | *Acer mandshuricum* Maxim. | 0.153 | 13.213 | 244.691 | 151.453 | 6.947 | 0.506 | 0.605 | 7.227 | 1.713 | 3.136 | 8.940 | 0.153 |
|  | *Betula platyphylla* Suk. | 0.165 | 27.067 | 202.696 | 145.385 | 6.680 | 0.416 | 0.571 | 1.289 | 0.626 | 3.370 | 1.915 | 0.165 |
|  | *Prunus cerasifera* f. *atropurpurea* | 0.190 | 19.793 | 141.897 | 196.667 | 5.874 | 0.531 | 0.583 | 8.449 | 2.429 | 2.858 | 10.878 | 0.190 |
|  | *Padus virginiana* ‘Canada Red’ | 0.186 | 25.963 | 147.073 | 171.880 | 5.536 | 0.524 | 0.681 | 12.971 | 3.860 | 3.429 | 16.831 | 0.186 |
|  | *Sorbus pohuashanensis* | 0.114 | 13.677 | 406.543 | 307.692 | 9.173 | 0.537 | 0.594 | 0.666 | 0.307 | 1.796 | 0.973 | 0.114 |
|  | *Spiraea thunbergii* Bl. | 0.128 | 3.057 | 244.533 | 309.145 | 6.357 | 0.422 | 0.725 | 2.388 | 0.824 | 1.434 | 3.212 | 0.128 |
|  | *Spiraea x bumalda* cv.Gold Flame | 0.215 | 13.017 | 174.484 | 206.154 | 5.155 | 0.368 | 0.465 | 8.167 | 2.501 | 3.736 | 10.668 | 0.215 |
|  | *Berberis thunbergii* DC. | 0.242 | 15.867 | 127.546 | 85.299 | 4.250 | 0.309 | 0.500 | 7.333 | 1.340 | 2.614 | 8.673 | 0.242 |
|  | *Acer ginnala* Maxim. | 0.180 | 23.257 | 255.381 | 584.786 | 8.054 | 0.478 | 0.640 | 0.393 | 0.230 | 2.116 | 0.623 | 0.180 |
|  | *Physocarpus opulifolius* var.*luteus* | 0.164 | 12.750 | 295.139 | 211.282 | 6.375 | 0.495 | 0.564 | 2.222 | 0.279 | 2.161 | 2.501 | 0.164 |
|  | *Physocarpus opulifolius 'Summer Wine'* | 0.139 | 18.133 | 283.099 | 258.376 | 5.894 | 0.550 | 0.614 | 17.582 | 4.684 | 5.319 | 22.266 | 0.139 |
|  | *Parthenocissus quinquefolia* (L.) Planch. | 0.214 | 37.490 | 235.293 | 114.701 | 3.877 | 0.641 | 0.728 | 1.643 | 0.505 | 1.443 | 2.148 | 0.214 |
|  | *Parthenocissus tricuspidata* | 0.218 | 28.564 | 189.583 | 75.897 | 3.381 | 0.635 | 0.703 | 0.495 | 0.270 | 1.566 | 0.765 | 0.218 |
|  | *Vitis vinifera* L | 0.162 | 119.745 | 345.218 | 349.790 | 5.685 | 0.812 | 0.847 | 1.798 | 0.448 | 0.522 | 2.246 | 0.162 |
| Bozhou | *Acer pictum*Thunb. ex Murray | 0.134 | 38.796 | 189.741 | 449.520 | 4.942 | 0.634 | 0.449 | 5.367 | 2.165 | 1.461 | 7.532 | 0.134 |
|  | *Prunus cerasifera* f. *atropurpurea* | 0.144 | 9.580 | 154.676 | 359.700 | 3.325 | 0.648 | 0.416 | 7.392 | 1.951 | 2.569 | 9.343 | 0.144 |
|  | *Ginkgo biloba* L. | 0.189 | 19.376 | 116.233 | 234.810 | 1.436 | 0.847 | 0.309 | 1.216 | 0.359 | 0.566 | 1.575 | 0.189 |
|  | *Acer palmatum* Thunb. | 0.140 | 12.845 | 168.793 | 559.290 | 5.105 | 0.780 | 0.442 | 1.626 | 0.336 | 0.565 | 1.961 | 0.140 |
|  | *Loropetalum chinense* var. *rubrum* | 0.188 | 6.403 | 96.050 | 286.240 | 3.669 | 0.737 | 0.421 | 4.080 | 0.172 | 0.554 | 4.252 | 0.188 |
|  | *Nandina domestica* Thunb. | 0.199 | 3.146 | 77.105 | 210.610 | 1.943 | 0.790 | 0.481 | 1.991 | 0.217 | 0.787 | 2.208 | 0.199 |
|  | *Photinia × fraseri* Dress | 0.209 | 17.632 | 105.835 | 344.460 | 3.357 | 0.709 | 0.333 | 2.556 | 0.351 | 0.848 | 2.907 | 0.209 |
|  | *Parthenocissus tricuspidata* | 0.174 | 124.118 | 198.620 | 325.720 | 3.976 | 0.854 | 0.305 | 6.554 | 1.936 | 1.361 | 8.490 | 0.174 |
|  | *Vitis vinifera* L. | 0.165 | 31.128 | 203.169 | 418.880 | 4.255 | 0.774 | 0.230 | 9.037 | 1.488 | 1.141 | 10.525 | 0.165 |
